# scFv-based biologics in diabetes: from therapeutic potential to clinical prospects

**DOI:** 10.3389/fimmu.2026.1809126

**Published:** 2026-06-04

**Authors:** Shikun Ge, Luoxuan Wang, Yichen Huang, Huaizu Guo, Fuyao Wei, Pan Li, Siyuan Li, Qian Liu, Jin Xu, Yilei Xiao, Alberto Carlos Piress Dias, Lusha Ji

**Affiliations:** 1 Shandong Key Laboratory of Applied Technology for Protein and Peptide Drugs, School of Pharmaceutical Sciences and Food Engineering, Liaocheng University, Liaocheng, China; 2 State Key Laboratory of Macromolecular Drugs and Large-scale Preparation, School of Pharmaceutical Sciences and Food Engineering, Liaocheng University, Liaocheng, China; 3 Department of Biology, Centre of Molecular and Environmental Biology (CBMA), University of Minho, Braga, Portugal; 4 State Key Laboratory of Macromolecular Drugs and Large-scale Preparation, School of Pharmaceutical Sciences, Wenzhou Medical University, Wenzhou, China; 5 State Key Laboratory of Macromolecular Drugs and Large-scale Preparation, NMPA Key Laboratory for Quality Control of Therapeutic Monoclonal Antibodies, Shanghai Zhangjiang Biotechnology Co., Ltd, Shanghai, China; 6 Department of Neurosurgery, Liaocheng People’s Hospital Affiliated to Shandong First Medical University, Liaocheng, Shangdong, China

**Keywords:** avian-derived antibodies, diabetes mellitus, precision medicine, single-chain variable fragment (ScFv), therapeutic antibodies

## Abstract

The global epidemic of diabetes mellitus the limitations of current therapies-including waning efficacy, adverse effects, and inability to modify disease progression-have accelerated the shift from conventional pharmacotherapy toward biologic agents. This review examines the emergence of single-chain variable fragments (scFv) as a versatile and engineerable class of biotherapeutics for precision diabetes management. Distinguished from full-length monoclonal antibodies (mAbs) by their compact size, enhanced tissue penetration, and modular design, scFv enable precise targeting of both autoimmune and metabolic pathways underlying type 1 diabetes and type 2 diabetes. We summarize advances in scFv applications spanning immunomodulation (e.g., via CTLA-4 and PD-1) and metabolic regulation (e.g., through insulin-degrading enzyme and GLP-1 receptor). Furthermore, we highlight the unique value of avian-derived scFv in recognizing conserved epitopes and overcoming immune tolerance, along with engineering strategies-such as Fc fusion, PEGylation, and multispecific formatting-that enhance pharmacokinetics and therapeutic efficacy. The clinical success of antibodies like Teplizumab underscores the translational potential of antibody-based platforms. Looking forward, scFv-based biologics, particularly when integrated with humanized Fc domains and half-life extension technologies, offer a promising and customizable strategy for next-generation diabetes therapy, bridging innovative drug design with clinically meaningful metabolic and immune modulation.

## 1 Introduction

**Figure 1:**
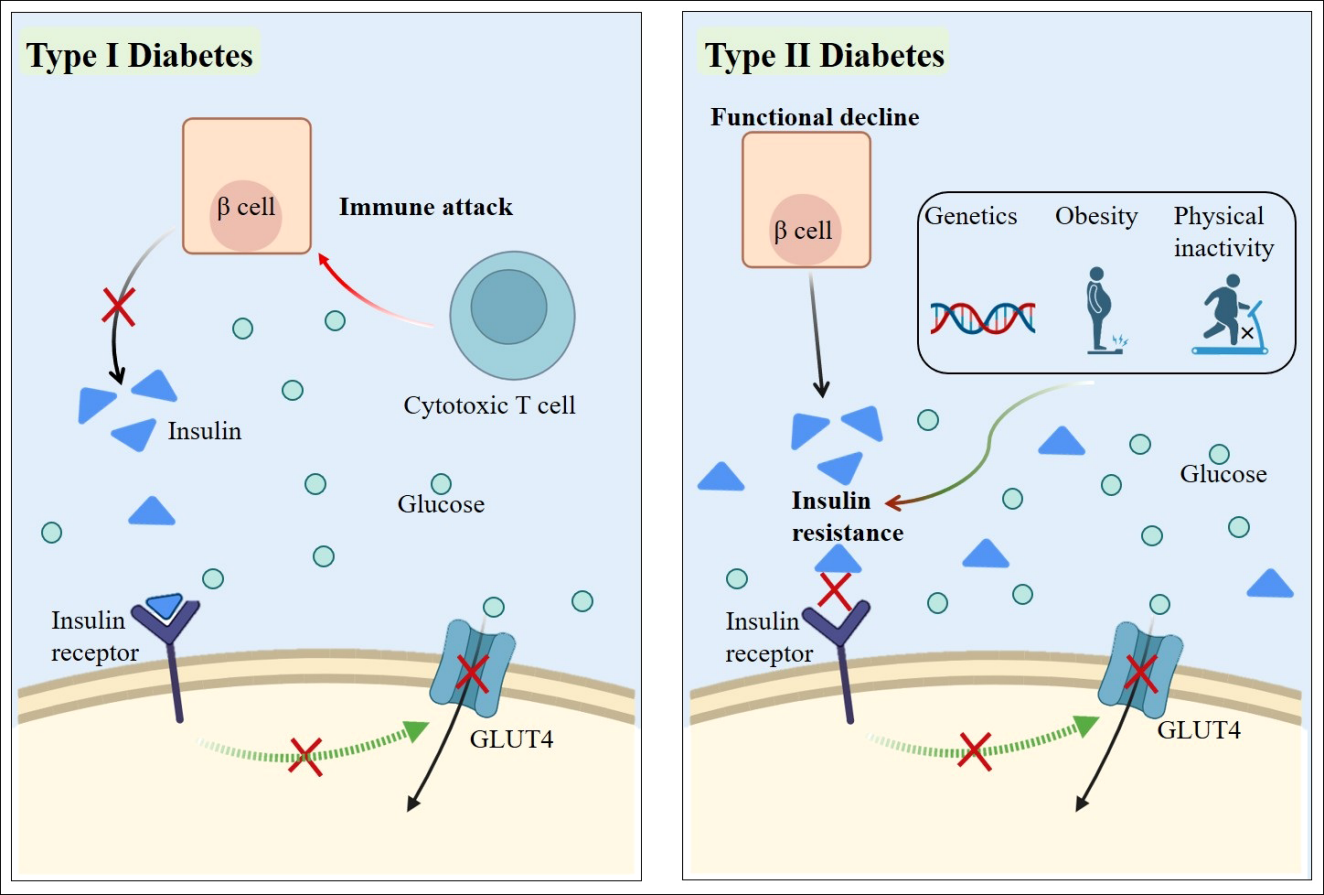
Comparison of the pathogenesis of type 1 and type 2 diabetes. Type 1 diabetes is caused by autoimmune-mediated destruction of pancreatic β-cells, resulting in absolute insulin deficiency, whereas type 2 diabetes arises from insulin resistance accompanied by a progressive decline in β-cell function. Consequently, the former is primarily immune-driven, while the latter is largely metabolic in origin (80–82).

**Figure 2:**
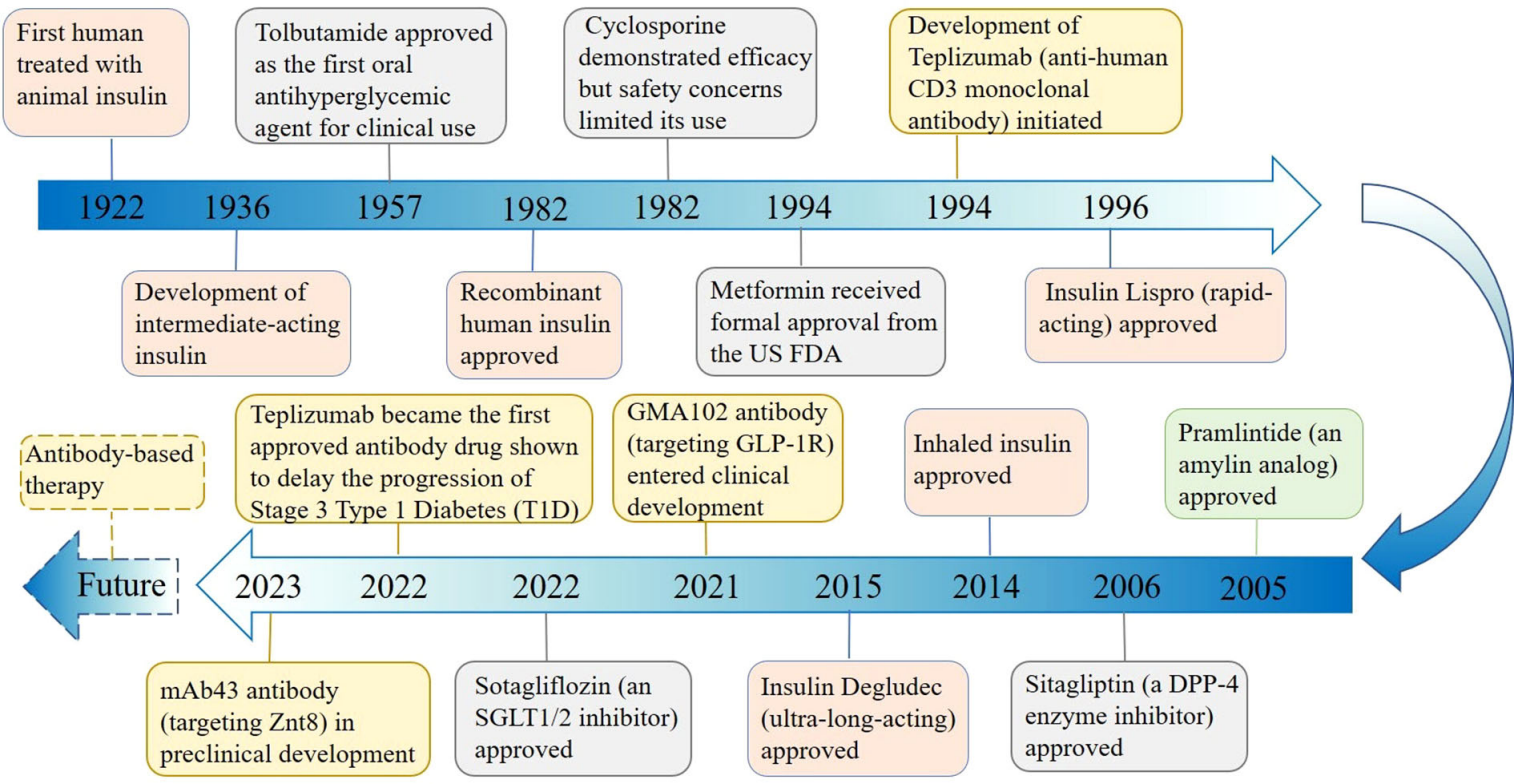
The development history of diabetes drugs. The development of pharmacological treatments for diabetes has progressed from the era of insulin replacement and oral sulfonylureas to engineered insulin analogs and incretin-based therapies, culminating recently in the approval of Teplizumab, a monoclonal antibody therapy for type 1 diabetes (83–86).

Diabetes mellitus (DM) stands as one of the most formidable global health challenges of the 21^st^ century, characterized by chronic hyperglycemia arising from defective insulin secretion, action, or both (**Figure 1**) (1). Its escalating prevalence fuels a massive burden of morbidity, mortality, and healthcare expenditure, primarily driven by debilitating microvascular and macrovascular complications (2–5). The therapeutic armamentarium has evolved considerably since the seminal discovery of insulin over a century ago (6). This was followed by oral agents such as metformin and sulfonylureas (7, 8), and more recently, by incretin-based therapies and sodium-glucose cotransporter-2 (SGLT-2) inhibitors, which offered improved glycemic control and cardiorenal benefits (9, 10) (**Figure 2**). Despite these advances, conventional pharmacotherapies predominantly manage hyperglycemia and are often limited by waning efficacy, side effects, and an inability to fundamentally arrest the underlying autoimmune attack in type 1 diabetes (T1D) or reverse the core metabolic dysfunction in type 2 diabetes (T2D) (9, 11, 12) (**Figure 1**). This critical gap underscores an urgent need for next-generation therapeutics capable of precise, mechanism-driven intervention (13, 14).

The emergence of biologic agents, particularly monoclonal antibodies (mAbs), heralded a paradigm shift toward targeted immunotherapy and metabolic intervention in diabetes (15, 16). Antibodies confer exceptional specificity for key pathogenic pathways. In T1D, agents like Teplizumab (anti-CD3) have clinically validated the principle of immunomodulation to preserve β-cell function and delay disease onset (15, 16). In T2D, antibody-based approaches are exploring metabolic regulators such as the GLP-1 receptor (17, 18). However, conventional full-length mAbs face their own limitations, including suboptimal tissue penetration, potential immunogenicity, and complex manufacturing, which can hinder their application, especially for targets within dense pancreatic islets or insulin-sensitive tissues (19).

These limitations have catalyzed the development of engineered antibody fragments, among which single-chain variable fragments (scFv) represent a particularly versatile and promising platform (20–22). Their small size (~25 kDa) facilitates superior tissue penetration and access to cryptic epitopes. Furthermore, the single-gene format of scFv enables unparalleled modular engineering. This includes affinity maturation, creation of bispecific/multispecific constructs to engage multiple targets simultaneously, and fusion with functional moieties (e.g., toxins, radionuclides, or effector Fc domains) for tailored therapeutic or diagnostic applications (23, 24).

A particularly innovative avenue is the derivation of scFv from avian species (e.g., chicken, ostrich) (25). The evolutionary distance between birds and mammals facilitates the generation of antibodies against highly conserved mammalian antigens, effectively bypassing immune tolerance barriers common in rodent immunizations. This approach has proven invaluable for generating functional antibodies against challenging targets like G-protein coupled receptors (GPCRs), yielding diverse epitope coverage and often potent antagonistic or agonistic activities (26). While scFv technology has been extensively applied and reviewed in fields like oncology (27–29), a dedicated synthesis of its multifaceted role in diabetes-spanning therapeutic mechanism, diagnostic innovation, and protein engineering-is notably lacking.

This review aims to provide a comprehensive analysis of scFv-based biologics in the context of diabetes. We will trace their development from a conceptual breakthrough to a burgeoning clinical prospect. The article will detail their applications in modulating immune checkpoints (e.g., CTLA-4, PD-1) in T1D and metabolic enzymes/receptors (e.g., insulin-degrading enzyme, GLP-1R) in T2D. We will highlight the unique advantages of avian-derived scFv libraries and critically evaluate molecular strategies designed to overcome the inherent pharmacokinetic (PK) limitations of scFv and enhance therapeutic efficacy. Finally, by integrating recent preclinical breakthroughs with pioneering clinical evidence, this review underscores the transformative potential of scFv-based platforms. We posit that these engineerable, precise biologics are poised to address the persistent therapeutic challenges across the autoimmune and metabolic spectrum of diabetes, bridging innovative molecular design with tangible clinical benefit.

## 2 Emergence of antibody-based therapeutics in diabetes management

**Figure 3:**
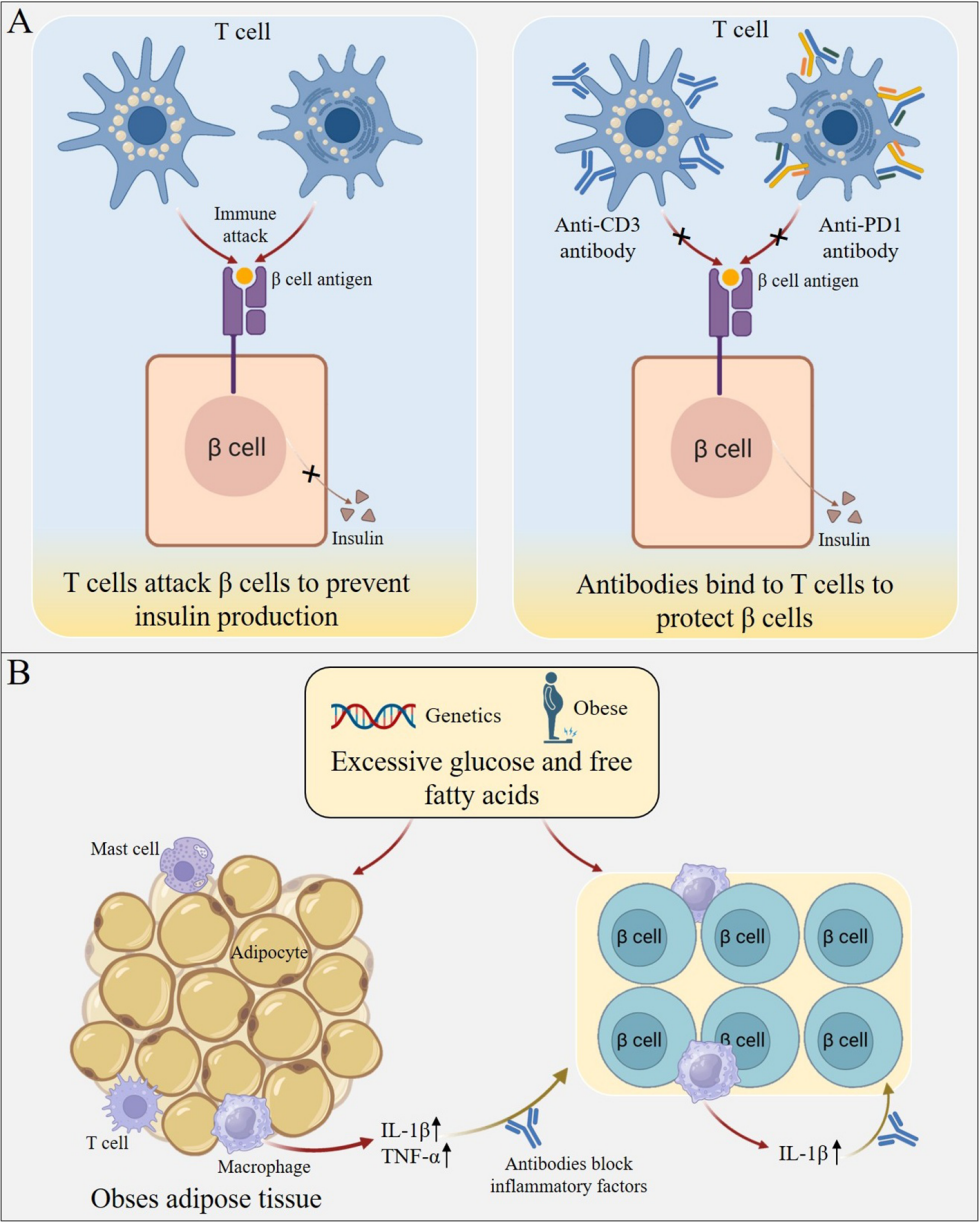
Mechanism of antibody-based therapies in diabetes. In T1D **(A)**, pancreatic β cells are selectively destroyed by autoreactive T cells that recognize β-cell-specific antigens, resulting in insulin deficiency. Therapeutic antibodies, such as anti-CD3, modulate T-cell activation and immune checkpoint pathways, thereby suppressing pathogenic immune responses and preserving β-cell viability (32, 33, 42). In T2D **(B)**, chronic exposure to excessive glucose and free fatty acids induces low-grade inflammation in adipose tissue and pancreatic islets through immune cell infiltration and increased secretion of pro-inflammatory cytokines, including IL-1β and TNF-α. Antibody-based therapies target these inflammatory mediators or their signaling pathways, thereby attenuating inflammatory responses, improving β-cell function, and restoring metabolic homeostasis (87–89).

The therapeutic management of diabetes has undergone a profound evolution, shifting from a historical focus on symptomatic glucose-lowering to a contemporary emphasis on mechanism-driven disease modification. This shift is exemplified by the rise of biologic agents, with mAbs at the forefront of a new therapeutic paradigm (30). The fundamental advantage of antibody-based therapies lies in their unparalleled specificity, enabling precise engagement of disease-driving pathways-autoimmunity in T1D and dysfunctional metabolic signaling in T2D-with minimal off-target effects (**Figure 3**) (15, 16).

In T1D, the clinical success of Teplizumab, an anti-CD3 mAb, marked a watershed moment. Its approval for delaying the onset of clinical T1D validated the principle of immunomodulation as a viable strategy to preserve residual *β*-cell function (31, 32). This approach moves beyond insulin replacement to directly intervene in the autoimmune cascade (10). Other antibody targets in T1D exploration include cytokines and co-stimulatory molecules aimed at re-establishing immune tolerance (33, 34). In T2D and obesity, antibody strategies are increasingly targeting key metabolic regulators. Beyond the established GLP-1 receptor agonists, research focuses on dual GIP/GLP-1 receptor co-agonists, antagonists of pathways like myostatin/activin to enhance muscle metabolism, and antibodies targeting inflammatory cytokines implicated in insulin resistance (17, 18, 35).

These therapies often possess favorable PK profiles with less inter-patient variability compared to small molecules, aligning with the goals of personalized medicine (15). However, conventional full-length mAbs are not without limitations. Their large size (~150 kDa) can hinder deep tissue penetration into pancreatic islets or adipose tissue. Furthermore, the potential for immunogenicity, complex manufacturing processes, and the presence of an Fc domain that may elicit unwanted effector functions in certain contexts present challenges (19). These limitations have catalyzed the development of next-generation antibody formats, setting the stage for the emergence of smaller, more engineerable fragments like the scFv as promising tools for overcoming these translational hurdles.

## 3 The scFv platform: design, engineering, and versatility

**Figure 4:**
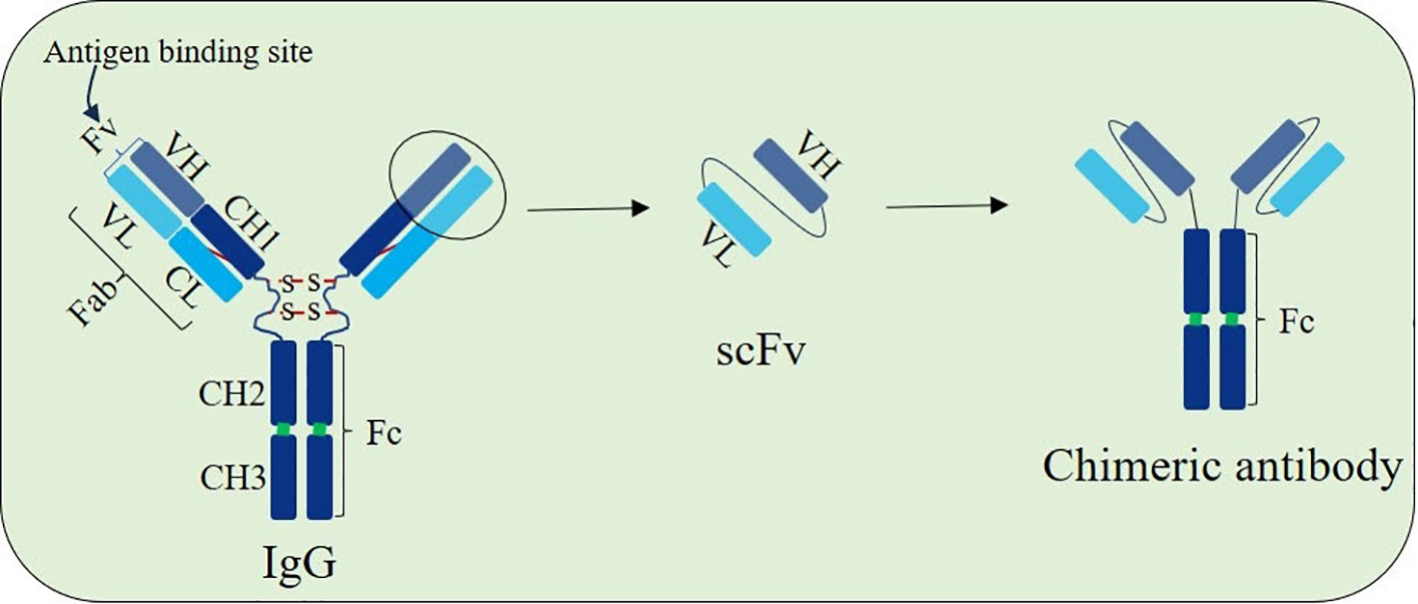
The scFv format of an antibody.

scFv represent a pivotal innovation in antibody engineering. Conceived in the late 1980s, an scFv is a minimal antigen-binding unit comprising the variable heavy (VH) and variable light (VL) chains of an antibody, tethered together by a flexible linker (**Figure 4**) (20–22). This elegant design strips away the constant regions (Fc) of a full-length antibody, reducing the molecular weight to approximately 25–30 kDa.

This compact structure confers several intrinsic advantages. The reduced size significantly enhances tissue penetration and tumor or islet access, a property extensively leveraged in oncology for imaging and therapy (27–29). scFvs also exhibit faster blood clearance, which is beneficial for diagnostic imaging but a challenge for therapeutic applications requiring sustained exposure. Critically, their recombinant, single-gene format unlocks extraordinary modularity for protein engineering (23). This allows for sophisticated customization to optimize function: Affinity maturation can be performed *in vitro* to generate scFvs with picomolar binding affinities. Multivalency and multispecificity can be engineered by linking two or more scFvs in tandem to simultaneously engage multiple epitopes or targets, enhancing avidity and enabling novel mechanisms of action; Functional fusion is readily achieved by genetically fusing scFvs fusion to toxins, radionuclides, or enzyme domains, creating targeted therapeutic or diagnostic conjugates (24).

The clinical translatability of scFv-based architectures has been exemplified by blinatumomab, the first FDA-approved bispecific T-cell engager, which consists of tandemly linked anti-CD19 and anti-CD3 scFvs. Its success in hematologic malignancies demonstrates how the modularity of scFv formats can be leveraged to enable multispecific targeting and clinically meaningful therapeutic efficacy, thereby validating scFv as a mature and engineerable therapeutic platform beyond conventional monoclonal antibodies (36, 37).

While scFv technology was pioneered and matured in oncology, its unique combination of small size, high specificity, and unparalleled engineering flexibility makes it an ideal, yet underexplored, platform for addressing the complex, compartmentalized pathologies of diabetes, from infiltrated pancreatic islets to insulin-resistant tissues (38–40).

## 4 Therapeutic and diagnostic application of scFv in diabetes

The application of scFvs in diabetes is expanding across both therapeutic and diagnostic frontiers, capitalizing on their unique properties to address specific challenges in T1D and T2D (**Table 1**).

**Table 1:** Representative studies of scFv in diabetes.

Target	Antibody species origin	Research model	Research objective	Efficiency	References
PTPRN extracellular domain	Ostrich	NA	Diagnosis and treatment for T1D	scFv exhibited a high affinity with *β* cells.	(47)
Human HbA1c	Sheep	NA	Diagnosis	scFv didn’t recognize glycated *β* Val-1 but was specific for HbA1c with high affinity.	(44)
TSPAN7	Human	HEK293T cells transfected with TSPAN7	Treatment for T1D	scFv showed favorable and strong binding patterns in flow cytometry.	(90)
PD-1	Mouse	NOD mice	Treatment for T1D	scFv improved RR-EAE in mice and no significant hepatotoxicity was observed.	(42)
IDE	Human	Streptozotocin-induced diabetic mice	Treatment for T2D	scFv bound human and mouse IDE with high affinity and specificity and significantly reduced blood glucose levels in mice.	(43)
GLP-1R	Human	NA	Treatment for T2D	The affinity of scFv obtained by a purified biochemical system for the antigen was increased 3-4-fold compared to the parental scFv.	(18)
GLP-1R	Human	C57/Bl6 mice	Treatment for T2D	scFv as an antagonistic antibody of GLP1R, binds to GLP1R on pancreatic *β*-cells and blocks the actions of GLP-1 *in vivo*.	(17)
Adiponectin	NA	NA	Diagnosis for T2D	scFv exhibited specific binding to adiponectin and no cross-reactivity with the structurally most alike protein Cq1.	(46)
ZnT8	Human	NA	Diagnosis and treatment for T1D	scFv specifically bound ZnT8 N/C fusion protein and ZnT8 C terminal dimer with one Arg325Trp mutation.	(45)
Mouse CTLA-4	Hamster	*β* cell-specific anti-CTLA-4 scFv transgenic NOD mice	Treatment for T1D	The transgenic expression of anti-CTLA-4 scFv on pancreatic *β* cells significantly protected NOD mice from spontaneous autoimmune diabetes.	(41)

CTLA-4, cytotoxic T-lymphocyte associated protein 4; GLP-1R: glucagon-like peptide-1 receptor; HbA1c, haemoglobin A1c; IDE: insulin-degrading enzyme; NA, not analysed; NOD mice, non-obese diabetic mice; PD-1, programmed death-1; PTPRN, protein tyrosine phosphatase receptor N; TSPAN7, tetraspanin 7; RR-EAE, relapsing-remitting experimental autoimmune encephalomyelitis; T1D, type 1 diabetes. ZnT8: zinc transporter 8.

### 4.1 Therapeutic application

In T1D, scFvs are being engineered for immunomodulation. Transgenic expression of a targeting CTLA-4 scFv specifically on pancreatic β-cells in NOD mice provided localized immune checkpoint blockade, significantly protecting against autoimmune diabetes without systemic toxicity (41). Similarly, scFv-based constructs targeting PD-1 have shown efficacy in autoimmune models (42). This approach enables precise immune modulation within the target organ. For T2D, scFvs targeting metabolic nodes are promising. A notable example is an scFv targeting IDE, which, by inhibiting IDE, stabilized endogenous insulin and significantly lowered blood glucose levels in diabetic mouse models (43). Antagonistic scFvs against the GLP-1R have also been developed as research tools to dissect receptor physiology (17). Furthermore, scFvs with agonistic potential for receptors like GLP-1R could offer an alternative to peptide-based agonists with potentially improved stability and half-life after engineering.

### 4.2 Diagnostic and imaging applications

The high specificity and small size of scFvs are ideal for developing sensitive diagnostics. They have been successfully selected against key biomarkers like glycated hemoglobin (HbA1c) for diabetes monitoring (44), autoantigens like ZnT8 for predicting T1D risk (45), and adiponectin for assessing metabolic health in T2D (46). Their potential extends to *in vivo* molecular imaging. Research models have utilized scFv to bind crucial disease markers such as the PTPRN extracellular domain (47), enabling early detection of insulitis or tracking of *β*-cell loss/gain in response to therapy. This capability remains a significant unmet need in diabetes research and clinical management.

Importantly, the clinical feasibility of monovalent scFv therapeutics has been validated by brolucizumab, the first FDA-approved scFv for neovascular retinal diseases, including diabetic retinopathy-related complications. Owing to its compact molecular size, brolucizumab achieves high molar dosing and enhanced tissue penetration in ocular tissues, highlighting the translational advantages of scFv-based therapeutics while simultaneously underscoring the importance of optimizing pharmacokinetic behavior and safety profiles for broader clinical applications (48).

## 5 Harnessing avian immune systems: avian-derived scFv for challenging targets

**Figure 5:**
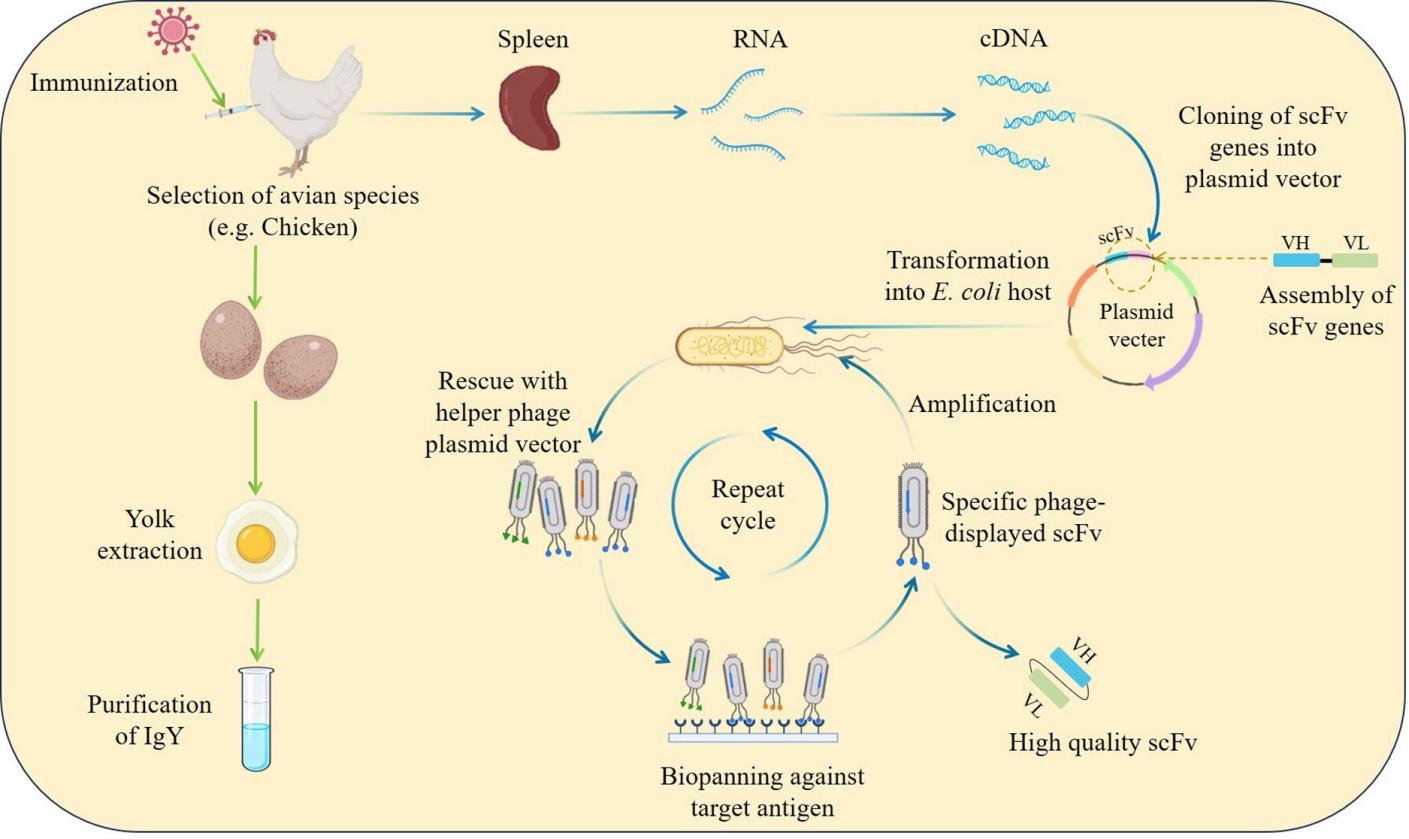
The generation of avian antibodies.

A powerful strategy to overcome the limitations of conventional rodent immunization, particularly for highly conserved mammalian targets, is the use of avian species (e.g., chickens, ostriches) as hosts for antibody generation (**Figure 5**). Due to their greater phylogenetic distance from mammals, avian immune systems are less constrained by mammalian self-tolerance mechanisms, enabling the generation of antibodies against highly conserved or weakly immunogenic mammalian epitopes that may not efficiently elicit immune responses in conventional mammalian hosts. This evolutionary divergence broadens the accessible antibody repertoire and may facilitate the development of scFvs targeting structurally conserved diabetes-associated antigens. However, additional humanization strategies may be required to reduce potential immunogenicity and improve clinical translatability (25, 26).

This approach has proven exceptionally valuable for generating functional antibodies against GPCRs, which are notoriously difficult targets due to their complex membrane topology and high sequence conservation across mammals. For instance, immunization of chickens against the human Glucose-dependent Insulinotropic Polypeptide Receptor (GIPR) yielded a diverse panel of scFvs, with a high proportion exhibiting potent antagonistic activity-a feat rarely achieved with mouse immunizations (26). Similarly, targeting the human glucose transporter GLUT4, which shares 95% identity with mouse GLUT4, is challenging in rodents. Chickens, whose GLUT homologs share only ~65% identity with human GLUT4, provide a viable path to generate specific antibodies without cross-reactivity issues (49).

Avian antibodies also offer simplified genetics, as they are derived from a single VH and VL germline family, streamlining library construction and subsequent humanization processes (49–51). Recent work has demonstrated the generation of an ostrich-derived scFv library and the successful isolation of high-affinity scFvs against the extracellular domain of PTPRN (47, 52), a *β*-cell antigen, showcasing the practical utility of this platform for diabetes target discovery (47). Thus, avian-derived scFv libraries serve as a rich and often essential resource for discovering functional binders against evolutionarily conserved, therapeutically relevant diabetes targets.

Despite their advantages in recognizing evolutionarily conserved mammalian epitopes, avian-derived scFvs may also present an increased risk of immunogenicity due to their greater phylogenetic divergence from human antibody repertoires (53). Therefore, additional humanization and framework optimization strategies may be required to facilitate their future clinical translation and improve biosafety profiles. An important alternative scaffold to scFv is the camelid-derived single-domain antibody (VHH or nanobody), which has emerged as a clinically validated antibody format with favorable developability characteristics (54). Compared with scFvs, VHHs are smaller, structurally simpler, and generally exhibit improved thermodynamic stability and reduced aggregation propensity due to the absence of VH-VL pairing and linker dependence (55, 56). Importantly, llama-derived VHHs are also highly suitable for generating binders against evolutionarily conserved mammalian epitopes and difficult membrane proteins such as GPCRs (57). Nevertheless, scFvs retain distinct advantages in modular engineering, including facile bispecific formatting and compatibility with established antibody architectures. Thus, VHHs and scFvs may be viewed as complementary rather than competing scaffolds, with scaffold selection depending on target biology, developability requirements, and translational objectives.

## 6 Engineering strategies to optimize scFv performance for clinical translation

The translation of scFv from a research tool to a viable clinical therapeutic is contingent upon overcoming its inherent PK limitations, primarily its rapid renal clearance leading to a short plasma half-life (often minutes to a few hours). A suite of sophisticated protein engineering strategies has been developed to address this and enhance overall drug-like properties.

scFvs inherently face stability and aggregation challenges arising from flexible linker design, VH/VL mispairing, and framework instability. Studies have shown that linker length and domain orientation critically influence folding and aggregation propensity, whereas alternative formats or framework engineering can markedly improve thermodynamic stability, manufacturability, and resistance to unfavorable intermolecular interactions (58, 59).

### 6.1 Half-life extension

The most common strategy is Fc fusion, creating an scFv-Fc dimer. The human Fc domain engages the neonatal Fc receptor (FcRn), facilitating cellular recycling and extending plasma half-life to days or weeks, akin to a full IgG (60). Amino acid substitutions within the Fc region have emerged as an effective strategy to improve the pharmacokinetic properties of scFv-Fc fusion proteins by enhancing FcRn-mediated recycling. Fc-engineered variants such as YTE (M252Y/S254T/T256E) and LS (M428L/N434S) have been shown to increase pH-dependent FcRn binding under acidic conditions, thereby prolonging serum half-life and improving *in vivo* therapeutic exposure (61, 62). In addition, Fc mutations such as LALA or PGLALA may attenuate Fcγ receptor and complement interactions, enabling optimization of immune effector functions while maintaining favorable pharmacokinetics (63, 64). Fc fusion improves half-life, but may not fully recapitulate IgG-like PK due to structural differences affecting FcRn interactions (65–67).

Human serum albumin (HSA) also represents an important endogenous scaffold for half-life extension of biologics through FcRn-mediated recycling. Notably, the clinical success of long-acting GLP-1 receptor agonists partly relies on lipid conjugation-mediated albumin binding, suggesting that albumin-based strategies may similarly enhance the pharmacokinetic performance of scFv therapeutics in diabetes (48). Alternative chemical approaches, such as PEGylation or site-directed glycosylation, further stabilize the scFv construct by conferring resistance against proteolytic degradation and reducing susceptibility to immune clearance mechanisms (68, 69).

### 6.2 Multispecific engineering

The modularity of scFv allows for the creation of bispecific or multispecific formats allows for the simultaneous engagement of multiple, distinct targets implicated in the pathogenesis of diabetes. This enables novel mechanisms of action beyond simple blockade or agonism (59).

### 6.3 Stability and affinity optimization

*In vitro* display technologies (phage, yeast, ribosome) coupled with directed evolution allow for affinity maturation to achieve sub-nanomolar binding. Furthermore, framework engineering can improve thermodynamic stability, reduce aggregation and increase production yield-critical for manufacturing (70–73).

Beyond affinity and pharmacokinetic optimization, rational engineering of scFv-based conjugates may further improve therapeutic efficacy and translational potential. Strategies including site-specific payload conjugation, linker tuning, and modular multivalent architectures have been explored to enhance drug loading capacity while minimizing disruption of antigen recognition and molecular integrity (59, 74). Meanwhile, risk management considerations, including mitigation of off-target binding and systemic toxicity through affinity modulation, target-restricted delivery, and controlled immune activation, are increasingly recognized as important parameters in the clinical development of scFv-based therapeutics (59).

These engineering efforts transform the scFv from a minimal binding fragment into a tunable therapeutic scaffold with tailored PK, stability, and functional profiles, paving the way for its clinical application in chronic diseases like diabetes.

## 7 From bench to bedside: chimeric and humanized antibodies in the clinical landscape

The clinical success story of antibody therapy in diabetes is currently authored by full-length, humanized mAbs (**Table 2**). Teplizumab, a humanized anti-CD3 mAb with engineered Fc mutations to reduce cytokine release, stands as the pioneering agent. Its approval for delaying Stage 3 T1D provides definitive proof-of-concept that immunomodulation can alter the disease course, protecting *β*-cells function (75, 76). Other anti-CD3 antibodies like Otelixizumab have shown dose-dependent effects in new-onset T1D (77). Beyond T-cell targets, the anti-CD20 antibody Rituximab (a chimeric mouse/human antibody) demonstrated in studies that B-cell depletion could modulate monocyte function and inflammation in recent-onset T1D, highlighting broader immune pathways (78). While not yet approved for diabetes, repurposed antibodies like those targeting IDE have been explored for their potential to reduced blood glucose levels (43).

**Table 2:** Summary of clinical antibody therapies in diabetes.

Target	Cross-species chimeric antibody	Research model	Research objective	Efficiency	References
CD3	Mouse anti-CD3 CDR was grafted into a human IgG backbone, and two mutations were introduced to Fc that do not bind to Fc receptors.	Clinical trials on humans	Treatment for T1D	^a^Teplizumab significantly delayed the onset of stage 3 T1D in adult and pediatric relatives of patients with stage 2 T1D in the event driven phase 2 TN-10 trial (NCT01030861).	(75, 91)
CD3	Humanized γ1 from the rat heavy chain. light chain λ is a rat variable region linked to the human constant region.	Patients aged 16–27 diagnosed with T1D in less than 32 days	Treatment for T1D	18 mg was the maximum tolerated dose of Otelixizumab and 9 mg was identified as the dose with the better therapeutic index.	(77)
CD20	Mouse variable region fusion with a human constant region.	Monocytes were obtained from recent-onset T1D patients.	Treatment for T1D	^b^Rituximab can reverse the abnormal functional activities of monocytes as well as their production of proinflammatory cytokines at the onset of T1D.	(78)
GCGR	A humanized murine antibody.	Adult patients aged 18–55 years who have been diagnosed with clinical-stage type 1 diabetes.	Treatment for T1D	Clinical data of Volagidemab demonstrated key outcomes, including a reduction in insulin dosage in the treatment group, without an increase in hypoglycemic events.	(92)
GIPR	Fully human antibody	Adults aged 18–65 years with obesity or overweight, with or without type 2 diabetes.	Treatment for T2D	Clinical data of AMG 133 showed potent dose-dependent weight loss (with sustained weight reduction for several months after a single dose) and a favorable safety profile.	(93)

^a^Teplizumab was approved by FDA in November 2022. ^b^Rituximab has been approved by the FDA for the treatment of non-Hodgkin’s lymphoma, but has not been approved for T1D. CD3, cluster of differentiation 3; CDR, complementarity determining region; IP, intraperitoneal; ITT, insulin tolerance test.

This clinical landscape sets the stage for the next wave: engineered scFv-based therapeutics. The scFv-Fc format represents a strategic middle ground, merging the benefits of scFv (small size for penetration, facile engineering of the binding domain) with the favorable PK and potential effector functions of an Fc domain. Avian-derived scFvs, once humanized and fused to a human Fc, create a cross-species chimeric platform that combines high-affinity, unique epitope recognition with human-compatible structure and extended half-life (60). Preclinically, chimeric antibodies targeting IDE have demonstrated metabolic benefits (43).

Recent advances in cell-based therapies have further expanded the therapeutic landscape for diabetes. A landmark first-in-human study demonstrated that genetically engineered allogeneic β cells could survive and maintain glucose-responsive insulin secretion in a patient with long-standing T1D without systemic immunosuppression. The transplanted donor-derived islet cells were modified using CRISPR-Cas12b-mediated gene editing and lentiviral transduction to evade immune rejection, and no significant immune response or treatment-related serious adverse events were observed during follow-up (79). Although this approach is distinct from antibody-based therapies, it highlights the growing convergence of immune engineering, cell therapy, and precision biologics in the pursuit of durable disease-modifying strategies for diabetes. These findings may also provide future opportunities for combining engineered β-cell replacement approaches with targeted antibody or scFv-based immunomodulatory therapies to further improve graft survival and immune tolerance.

The future clinical pipeline will likely feature such optimized constructs. They offer the potential for greater tissue penetration than full mAbs, the ability to target conserved epitopes via avian sequences, and the flexibility to be formatted as bispecifics or drug conjugates. The clinical validation provided by Teplizumab and others de-risks the antibody approach for diabetes, creating a receptive environment for these next-generation, scFv-driven biologics to transition from promising bench-top molecules to bedside therapies.

## 8 Conclusions and future perspectives

The evolution from the “insulin era” to the “targeted biologic era” is well underway. Engineered fragments like scFv and scFv-Fc chimeras are not merely smaller versions of mAbs; they are customizable tools that solve specific structural and biological problems inherent to diabetes pathology. By combining the unique epitope recognition of avian immune systems with advanced humanization and half-life extension technologies, the next generation of diabetes therapeutics will likely offer greater efficacy, reduced immunogenicity, and the capacity to precisely modulate the complex interface between metabolism and immunity.
