# Correction: scFv-based biologics in diabetes: from therapeutic potential to clinical prospects

**DOI:** 10.3389/fimmu.2026.1920181

**Published:** 2026-07-13

**Authors:** Shikun Ge, Luoxuan Wang, Yichen Huang, Huaizu Guo, Fuyao Wei, Pan Li, Siyuan Li, Qian Liu, Jin Xu, Yilei Xiao, Alberto Carlos Piress Dias, Lusha Ji

**Affiliations:** 1 Shandong Key Laboratory of Applied Technology for Protein and Peptide Drugs, School of Pharmaceutical Sciences and Food Engineering, Liaocheng University, Liaocheng, China; 2 State Key Laboratory of Macromolecular Drugs and Large-scale Preparation, School of Pharmaceutical Sciences and Food Engineering, Liaocheng University, Liaocheng, China; 3 Department of Biology, Centre of Molecular and Environmental Biology (CBMA), University of Minho, Braga, Portugal; 4 State Key Laboratory of Macromolecular Drugs and Large-scale Preparation, School of Pharmaceutical Sciences, Wenzhou Medical University, Wenzhou, China; 5 State Key Laboratory of Macromolecular Drugs and Large-scale Preparation, NMPA Key Laboratory for Quality Control of Therapeutic Monoclonal Antibodies, Shanghai Zhangjiang Biotechnology Co., Ltd, Shanghai, China; 6 Department of Neurosurgery, Liaocheng People’s Hospital Affiliated to Shandong First Medical University, Liaocheng, Shangdong, China

**Keywords:** avian-derived antibodies, diabetes mellitus, precision medicine, single-chain variable fragment (ScFv), therapeutic antibodies

Affiliation 1 was erroneously written as “State Key Laboratory of Macromolecular Drugs and Large-scale Preparation, School of Pharmaceutical Sciences and Food Engineering, Liaocheng University, Liaocheng, China”. It has been corrected to read “Shandong Key Laboratory of Applied Technology for Protein and Peptide Drugs, School of Pharmaceutical Sciences and Food Engineering, Liaocheng University, Liaocheng, China”. 

Affiliation 2 was erroneously written as “State Key Laboratory of Macromolecular Drugs and Large-scale Preparation, School of Pharmaceutical Sciences, Wenzhou Medical University, Wenzhou, China”. It has been corrected to read “State Key Laboratory of Macromolecular Drugs and Large-scale Preparation, School of Pharmaceutical Sciences and Food Engineering, Liaocheng University, Liaocheng, China”. 

Affiliation 3 was erroneously written as “State Key Laboratory of Macromolecular Drugs and Large-scale Preparation, National Medical Products Administration (NMPA) Key Laboratory for Quality Control of Therapeutic Monoclonal Antibodies, Shanghai Zhangjiang Biotechnology Co., Ltd, Shanghai, China”. It has been corrected to read “Department of Biology, Centre of Molecular and Environmental Biology (CBMA), University of Minho, Braga, Portugal”. 

Affiliation 4 was erroneously written as “Department of Neurosurgery, Liaocheng People’s Hospital Affiliated to Shandong First Medical University, Liaocheng, Shangdong, China”. It has been corrected to read “State Key Laboratory of Macromolecular Drugs and Large-scale Preparation, School of Pharmaceutical Sciences, Wenzhou Medical University, Wenzhou, China”. 

Affiliation 5 was erroneously written as “5Department of Biology, Centre of Molecular and Environmental Biology (CBMA), University of Minho, Braga, Portugal”. It has been corrected to read “State Key Laboratory of Macromolecular Drugs and Large-scale Preparation, NMPA Key Laboratory for Quality Control of Therapeutic Monoclonal Antibodies, Shanghai Zhangjiang Biotechnology Co., Ltd, Shanghai, China”. 

The affiliations for author Huaizu Guo were incorrect given as affiliations 1, 2 and 3. It has now been corrected to “2 State Key Laboratory of Macromolecular Drugs and Large-scale Preparation, School of Pharmaceutical Sciences and Food Engineering, Liaocheng University, Liaocheng, China”, “3 Department of Biology, Centre of Molecular and Environmental Biology (CBMA), University of Minho, Braga, Portugal” and “4 State Key Laboratory of Macromolecular Drugs and Large-scale Preparation, School of Pharmaceutical Sciences, Wenzhou Medical University, Wenzhou, China”. 

As a result of this, the Conflict of Interest statement has been correct to: “Author JX was employed by the company Shanghai Zhangjiang Biotechnology Co., Ltd. The remaining authors declare that the research was conducted in the absence of any commercial or financial relationships that could be construed as a potential conflict of interest”. 

Author “Shikun Ge” was erroneously assigned as corresponding author. The correct corresponding author is solely “Lusha Ji”. 

The Reference for “12” was erroneously written as “Gao H-O, Chen F, Wang S-O. Hesperidin reduces systolic blood pressure in diabetic patients and has no effect on blood pressure in healthy individuals: a systematic review and meta-analysis. (2024) 38(7):3706–19. doi: 10.1002/ptr.8231”. It should be “Gao H, Chen F, Wang S. Hesperidin Reduces Systolic Blood Pressure in Diabetic Patients and Has No Effect on Blood Pressure in Healthy Individuals: A Systematic Review and Meta-Analysis. Phytother Res (2024) 38(7):3706-19. Epub 2024/05/22. doi: 10.1002/ptr.8231”. 

The original version of this article has been updated. 

